# Smart‐Adhesive, Breathable and Waterproof Fibrous Electronic Skins

**DOI:** 10.1002/advs.202405828

**Published:** 2024-07-25

**Authors:** Di Tan, Xiaoyang Guan, King Yan Chung, Yun Tang, Yujue Yang, Qian Wang, Tiandi Chen, Bingang Xu

**Affiliations:** ^1^ Nanotechnology Center School of Fashion and Textiles The Hong Kong Polytechnic University Hung Hom 999077 Hong Kong

**Keywords:** breathability, electronic skin, strain sensing, switchable adhesion, waterproof

## Abstract

For the need of direct contact with the skin, electronic skins (E‐skins) should not only fulfill electric functions, but also ensure comfort during wearing, including permeability, waterproofness, and easy removal. Herein, the study has developed a self‐adhesive, detach‐on‐demand, breathable, and waterproof E‐skin (PDSC) for motion sensing and wearable comfort by electrospinning styrene‐isoprene block copolymer rubber with carbon black nanosheets as the sensing layer and liner copolymers of N, N‐dimethylacrylamide, n‐octadecyl acrylate and lauryl methacrylate as the adhesive layer. The high elasticity and microfiber network structure endow the PDSC with good sensitivity and high linearity for strain sensing. The hydrophobic and crystallizable adhesive layer ensures robust, waterproof, and detaching‐on‐demand skin adhesion. Meanwhile, the fiber structure enables the PDSC good air and water permeability. The integration of electric and wearable functions endows the PDSC with great potential for motion sensing during human activities as both the sensing and wearable performances.

## Graphical abstract

A Janus electronic skin (E‐skin) with self‐adhesive, detach‐on‐demand, breathable, and waterproof properties has been designed and fabricated with electrospinning. This E‐skin integrates wearability and electronic functions through a fibrous rubber/carbon black composite layer for strain sensing and linear polymerized semicrystalline acrylate fibers for self‐adhesion. The integrated electric and wearable functions offer the E‐skin great potential in motion sensing.

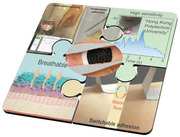

## 1 Introduction

Electronic skins (E‐skins), which are inspired by human skins, are developed as ideal platforms for areas like physiological monitoring, motion sensing, and Internet of Things owing to their flexibility, elasticity, light weight, biocompatibility, and skin conformability.^[^ ^1^, ^2^, ^3^, ^4^, ^5^, ^6^, ^7^ ^]^ The E‐skin strain sensors can work as intelligent extensions of human skins, converting mechanical deformations into electric signals for monitoring and analysis.^[^ ^5^, ^6^, ^7^, ^8^, ^9^, ^10^, ^11^, ^12^, ^13^, ^14^, ^15^, ^16^, ^17^ ^]^ As skin‐attaching devices, E‐skins need to fulfill not only sensing functions like sensitivity for different applications, but also wearable functions including self‐adhesion, breathability, waterproofness, and detachment‐on‐demand.^[^ ^6^, ^13^, ^14^, ^18^ ^]^ These wearable functions for comfort can greatly promote the long‐time and normalized wearing of E‐skins. The integration of wearable comfort and electronic functions become one of the most important directions of the next‐generation E‐skins.

Although plenty of E‐skins with good performance have been developed for body motion monitoring using different elastic materials like elastomers ^[^ ^7^, ^17^, ^19^, ^20^, ^21^, ^22^, ^23^, ^24^, ^25^ ^]^ and hydrogels,^[^ ^26^, ^27^, ^28^, ^29^, ^30^, ^31^, ^32^ ^]^ a majority of these E‐skins usually suffer from drawbacks in wearability like air and water permeability, which blocks sweat evaporation during daily activities, causing skin irritation and inflammation.^[^ ^14^, ^33^, ^34^ ^]^ Electrospinning provides a simple but effective way to fabricate E‐skins with inherent permeability for air and moisture. The fibrous structure can work as the air and moisture channels.^[^ ^16^, ^18^, ^34^, ^35^, ^36^, ^37^, ^38^, ^39^, ^40^, ^41^, ^42^, ^43^, ^44^, ^45^, ^46^, ^47^, ^48^ ^]^ The flexibility allows for the fabrication of e‐skins with diverse functionalities, like electrodes for physiological signal monitoring and motion sensing.^[^ ^34^, ^36^, ^37^ ^]^ However, these fibrous E‐skins usually lack specialized skin adhesion design, which mainly depends on van der Waals force from the low stiffness or extra tapes.^[^ ^12^, ^34^, ^35^, ^36^, ^37^, ^39^, ^40^, ^41^, ^42^, ^43^, ^44^, ^46^ ^]^ Van der Waals adhesion is weak and hard to support the long‐time wearing.^[^ ^12^, ^34^, ^36^, ^37^ ^]^ During daily activities, it is inevitable to expose E‐skins to water, especially sweat secretion between the skin and E‐skin interfaces, which can disable the van der Waals force. The commercial tapes are also hard to adhere to sweaty skin. The waterproof ability is one of the crucial wearable functions for long‐time and stable use.^[^ ^21^, ^35^, ^49^, ^50^, ^51^, ^52^ ^]^ E‐skins with durability with sweat and water can greatly extend the application scenarios like doing exercise and underwater activities. Even though the strong adhesion can ensure stable contact during wearing, the robust adhesion can lead to discomfort and even damage to the skin during the detachment after use.^[^ ^33^ ^]^ Detaching‐on‐demand can guarantee detaching comfort and safety after use, especially on the young and injured skins. Developing E‐skins with integrated strain sensing, breathability, waterproofing, and detach‐on‐demand ability is in high demand but also poses a significant challenge.

Here, we reported an E‐skin (PDSC) integrated with self‐adhesive, detach‐on‐demand, breathable, and waterproof functions for motion sensing and wearable comfort simultaneously. Styrene‐isoprene block copolymer rubber (SIS) with carbon black nanosheets (CB) was electrospun as the sensing layer. The soluble linear copolymer composed of N, N‐dimethylacrylamide (DMA), n‐octadecyl acrylate (C18A), and lauryl methacrylate (C12M) was prepared for electrospinning as the adhesive layer. The all‐fiber‐based structure endows the PDSC with good air and moisture permeability to ensure long‐time comfortable wearing. Meantime, the microscale SIS fiber network composited with conductive CB imparts sensitive and linear strain sensing to PDSC E‐skin, enabling tiny deformation detections like throat deformation by speaking. The crystallizable C18A segment in the adhesion layer provides ∼7 times stronger adhesion than commercial medical tapes and the inherent hydrophobicity of C18A and C12M segments guarantees the waterproof ability, thus ensuring stable and water‐durable attachment for a long time. Moreover, the crystallizable C18A segment also endows PDSC with detaching‐on‐demand ability to ensure easy and comfortable detaching after use. The integration of self‐adhesion, detaching‐on‐demand, breathability, and waterproof ability in the sensitive E‐skin strain sensor positions it as a highly promising tool for applications like motion sensing, healthcare, etc.

## 2 Result and Discussion

### 2.1 Fabrication and Basic Properties of PDSC E‐Skins

**Figure 1:**
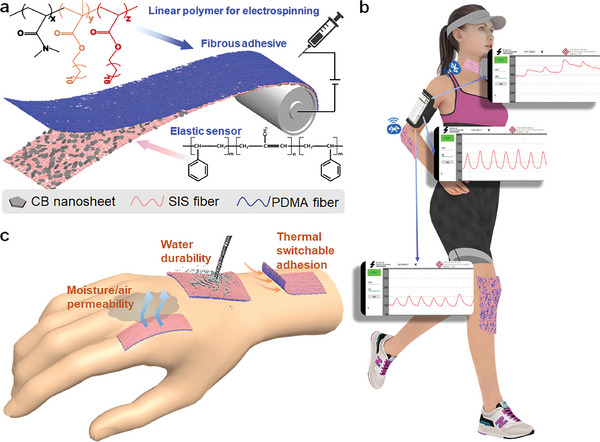
a) Electrospinning of the SIS/CB sensor layer and the PDMA adhesive layer. b) Illustration of PDSC E‐skin for real‐time motion monitoring. c) Illustration of PDSC E‐skin with breathability, waterproofness, and detaching‐on‐demand.

The Janus PDSC E‐skin comprises two different layers prepared both by electrospinning (Detailed in the Experimental section). The styrene‐isoprene block copolymer (SIS) rubber was electrospun with conductive carbon black (CB) nanosheets for the composite sensing layer (**Figure** **1**a). Then, the adhesive layer (PDMA) for skin adhesion was fabricated on the SIS/CB layer by electrospinning of copolymer of N, N‐dimethylacrylamide (DMA), n‐octadecyl acrylate (C18A) and lauryl methacrylate (C12M) (Figure 1a), which has been proved good biocompatibility by the culture of mouse embryonic fibroblasts (MEFs).^[^ ^21^ ^]^ For easy electrospinning, the PDMA copolymer was linearly polymerized without any crosslinker for solubility. The PDSC E‐skin is then obtained for real‐time strain sensing like motion detection, exercise monitoring, and so on (Figure 1b). The all‐fiber architecture also endows the PDSC with good air/moisture permeability, ensuring sustained comfort during prolonged wearing (Figure 1c). The hydrophobic PDMA adhesive layer offers the PDSC E‐skin good waterproofness to ensure stability in sweaty or watery conditions (Figure 1c). Meanwhile, by introducing the crystallizable C18A segment, the adhesion layer of PDSC can not only provide stable adhesion while in use, but also detach on demand by thermal after use, avoiding discomfort or damage to the skin (Figure 1c).

**Figure 2:**
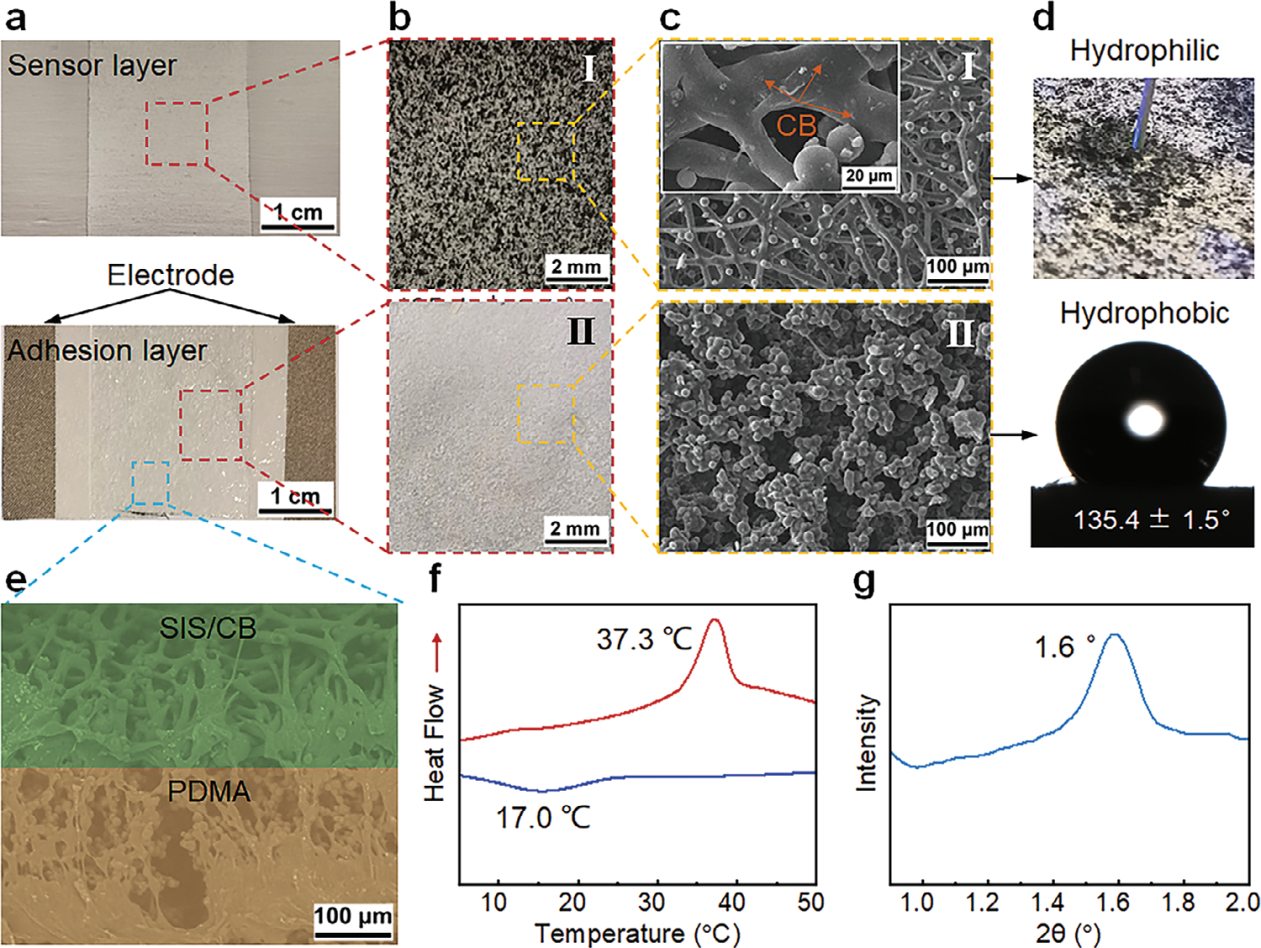
a) The photo of the PDSC E‐skin. b) The photos of the sensor layer (I) and the adhesion layer (II). c) SEM images of (I) sensor layer and (II) adhesion layer. d) Hydrophobicity of sensor layer and adhesion layer. e) The SEM image of cross‐section of PDSC. f) The DSC scan of PDSC E‐skin. g) The SAXS results of PDSC E‐skin.

The structure of PDSC is shown as **Figure** **2**a. The sensing layer exhibits black spots due to the compositing of conductive CB for resistance strain sensing while the adhesion layer composed of PDMA is white (Figure 2b). From the SEM images, there are distinct micro‐network structures in the sensor layer and adhesion layer (Figure 2c). The microscale fiber networks were formed by the electrospinning of SIS and electrospraying CB together. Because of the ethyl acetate (EA) solvent used in both SIS and CB solutions, CB nanosheets were merged into the SIS fibers. Only a minimal amount of CB nanosheets can be seen on the surface of SIS fibers with uniform distribution (Figure 2cI). The uniformly dispersed microspheres are PDMA, which infiltrated from the SIS/CB micro‐network during the electrospinning of the adhesive layer (Figure 2cI). The adhesion layer is composed of bead‐like fiber networks (Figure 2cII). The bead‐like structure was believed to result from the high surface energy and low viscosity of the PDMA/ethyl acetate (EA) solution. Meanwhile, the PDSC showed Janus hydrophobicity, which can benefit the moisture permeability when wearing.^[^ ^35^, ^53^, ^54^ ^]^ The sensor layer showed hydrophilic property as the water infiltrated into the SIS/CB layer right away while the adhesion layer showed the hydrophobicity of 135.4 ± 1.5 ° water contact angle as the hydrophobic C18A and C12M components (Figure 2d). From the cross‐section of PDSC, the SIS/CB sensor layer, PDMA adhesion layer, and the intimate interfaces as the same solvent, EA, was used for both the sensing and adhesive layer can be clearly seen (Figure 2e). Meanwhile, the Cu‐Ni cloth electrode was sandwiched between the sensing layer and adhesive layer during the fabrication process. The Cu‐Ni cloth electrode showed intimate combination with both the SIS/CB sensor layer and PDMA adhesion layer and have negligible influence to sensing and adhesion layers (Figure 2a; Figure S1a, Supporting Information).

The crystallization and melting of the C18A component are key to both robust adhesion and detaching‐on‐demand ability. With the proper content of C18A, the melting and crystallization temperature of the PDSC patch are adjusted to ≈37.3 and ≈17.0 °C, respectively (Figure 2f). Compared to a solid PDMA film with the same contents, the melting temperature is close while the crystallization temperature is lower (Figure S1b, Supporting Information), which may be ascribed to that the microstructures of PDMA layer fabricated by electrospinning have an obstructing effect on crystallization. By small‐angle X‐ray scattering measurement, a high‐intensity peak at 2θ = 1.6° is obtained (Figure 2g), indicating a long‐range ordering with a lattice spacing of ≈5.3 nm of C18A side chain crystals.^[^ ^21^, ^55^, ^56^ ^]^

### 2.2 Adhesion of PDSC E‐Skin

**Figure 3:**
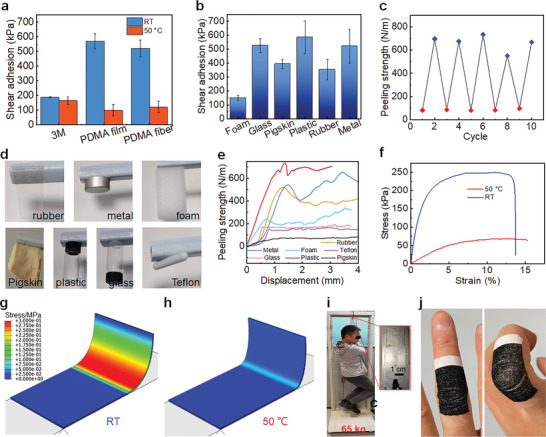
a) The shear adhesion strength of PDMA fiber, PDMA solid film and 3M double‐side tape on the steel. b) The shear adhesion of PDMA fiber on different substrates. c) The switchable peeling adhesion cycles of PDSC E‐skin. d) The photo and e) peeling strength of PDSC E‐skin on different kinds of surfaces. f) The tensile tests of PDSC at RT and 50 °C. g) The FEA simulation of PDSC at RT and h) 50 °C. i) The 2 × 2 cm^2^ PDSC bearing a 65 kg man. j) The conformal adhesion of PDSC E‐skin on the human skin.

To evaluate the self‐adhesion of PDSC E‐skin, both shear and peeling adhesion tests were conducted. A 10 × 20 mm^2^ single PDMA adhesion layer was used for the shear adhesion tests (Figure S2a, Supporting Information). Two steel plates were used as the substrates, and a semiconductor heater with 50 °C constant heating temperature was used to heat the adhesives (Figure S2b,c, Supporting Information). During the tests, the adhesives were heated to 50 °C first and brought into contact with the substrates. Then the shear adhesion forces were tested by a universal testing machine at room temperature (22 °C, RT) and 50 °C, respectively. The PDMA demonstrated a shear adhesion strength on the steel of 520.9 ± 37.9 kPa at RT, over 2 times higher adhesion than the commercial 3M double‐side tapes. Meanwhile, the shear adhesion of PDMA was switched off to ≈100 kPa at 50 °C, lower than the adhesion of 3M tapes (≈165 kPa) (**Figure** **3**a; Figure S3a, Supporting Information). Compared to a solid PDMA film, the electrospun PDMA fibers showed almost the same adhesion performance (Figure 3a), indicating the fiber structures have little effect on shear adhesion performance. Meanwhile, the electrospun PDMA fibers showed versatile adhesion on different substrates. The shear adhesion of PDSC on foam, glass, pigskin, plastic, and rubber reached ≈149, 526, 394, 585, and 352 kPa, respectively (Figure 3b; Figure S3b, Supporting Information).

In addition to shear adhesion, peeling adhesion, which is also important for E‐skins, was evaluated on a polyethylene terephthalate (PET) substrate with an electric heater as the temperature control table (Figure S4a,b, Supporting Information). Similar to the shear adhesion, the PDSC E‐skin also showed thermal switchable peeling adhesion compared to 3M commercial medical tape. At RT, the PDSC E‐skin exhibited a peeling adhesion strength of ≈700 N/m, almost seven times higher than the medical tapes (Figure 3c). However, when heating the PET substrate to 50 °C, the peeling adhesion is switched off to ≈85 N m^−1^ while the commercial medical tape maintains its peeling strength of ∼ 100 N/m (Figure 3c; Figure S4c, Supporting Information). And the repeated tests of peeling demonstrated the good reusability of PDSC E‐skin (Figure 3c). The superior adhesion of PDSC E‐skin compared to commercial tapes endows the PDSC E‐skin with excellent adaptability to different kinds of surfaces (Figure 3d). The peeling strength of PDSC on rubber, metal, foam, pigskin, Teflon, glass, and plastic reached ≈430, 486, 208, 80, 155, 166, and 680 N m^−1^, respectively (Figure 3e). Although the substrate roughness and surface energy do impact on the adhesive strength, the adhesive strength of PDSC is still higher than that of commercial tapes. The outstanding adaptability of PDSC adhesion is attributed to van der Waals interaction, which is mainly dependent on the contact state between PDSC and the substrates.^[^ ^21^ ^]^ When contact with the substrate, the PDSC was heated to ≈50 °C, at which point the modulus of PDSC decreased to ≈0.97 MPa. Consequently, the PDSC can form conformal contact even on rough and porous substrates, thus generating robust adhesion.

Meanwhile, the adjustment of modulus through thermal input endows PDSC with adhesion switchability for both shearing and peeling conditions. When shearing, the maximum sustainable adhesion force, *F*_c_, can be defined as follows ^[^ ^57^ ^]^:

(1) $$F_{c}\;∼\;\sqrt{G_{c}}\sqrt{\frac{AE}{C}}$$

where *G*_c_ is the critical strain energy release rate, which is dominated by the adhesion work. *A* is the contact area, *C* is the geometry parameter and *E* is the elastic modulus of E‐skin. Whether the adhesion is on or off, the *A*, *C*, and *G*_c_ are basically the same as the initial contact states are the same. Therefore, when the *E* of PDSC decreased from 17.3 MPa at RT to 0.97 MPa at 50 °C, the adhesion of PDSC had a ≈5 times change (Figure 3f,c). Similarly, during peeling, the work required for peeling (*W*_p_) is derived mainly from two components: the decohesion energy (*W*_ad_) and the potential energy of the tapes (*W*_po_): ^[^ ^58^ ^]^

(2) $$W_{p}=W_{ad}+W_{po}$$

The *W*_ad_s of PDSC at RT and 50 °C are presumed to be the same as the same attaching process. However, the high modulus at RT can greatly increase the *W*_po_ of PDSC, thus having the high energy dissipation of peeling adhesion. By finite element analysis (FEA), the bearing stress of PDSC peeling zone at RT reached a maximum stress of 322.5 kPa. However, at 50 °C, the stress at the peeling zone of PDSC is 81.3 kPa, only 25.2% of that at RT (Figure 3g,h). The high bearing stress at the peeling zone means the high energy dissipation under peeling, thus having the high peeling adhesion strength at RT. The strong adhesion allows a 20*20 mm^2^ PDSC E‐skin to support an adult with 65 kg weight (Figure 3i). Meanwhile, the PDSC E‐skin can form conformal contact with rough human skin, even with dense and deep skin folds, guaranteeing the stable functionality as E‐skins (Figure 3j).

### 2.3 The Sensing Function of PDSC E‐Skin

**Figure 4:**
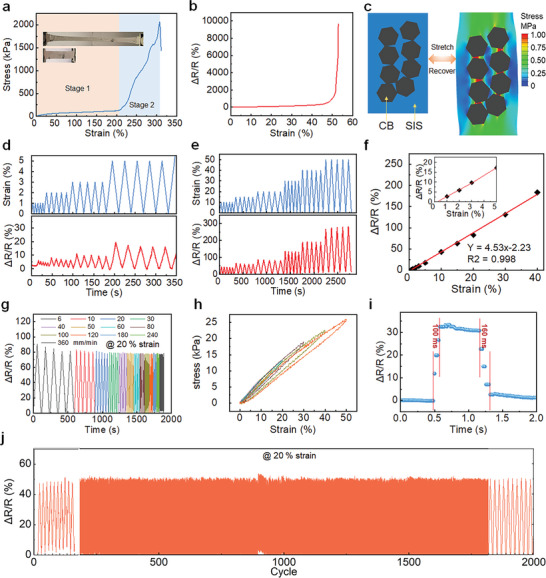
a) The tensile test of the PDSC E‐skin. b) The *ΔR*/*R* during the tensile tests. c) The FEA results for illustration of strain sensing mechanism. d)The small strain sensing test and relevant *ΔR*/*R* signals. e) The large strain sensing test and relevant *ΔR*/*R* signals. f) The linear fitting of *ΔR*/*R* and strain. g) The sensing tests at different stretching speeds under 20% strain. h) The hysteresis tests of PDSC under different strains. i) The response time of PDSC during stretching and recovery. j) The durability of PDSC E‐skin under 2000 cycles under 20% strain.

Using SIS as the base material endows the PDSC E‐skin with good tensile properties. The whole stretching process can be divided into two stages. The stage 1 is the deformation of the fiber network structures, in which the stress increases slowly with the strain until the strain reaches 200% (**Figure** **4**a). Then the stress increases dramatically when the strain exceeds 200%. At this stage, the deformation of the fiber structure is basically complete, so the main deformation comes from the elastic deformation of the SIS material itself rather than the fibrous structure (Figure 4a). The unsmooth stress‐strain curve of PDSC E‐skin in this stage is mainly due to the rough edges when cut. The cut fibers are the weak spots of the sample with the discontinuity of stress during the stretching. After breakage, the tensile strength and elongation at break of PDSC E‐skin can reach ≈2000 kPa and ≈300%, respectively (Figure 4a). The carbon black (CB) in the SIS fibers forms the conductive network. During the tension, the relative resistance change (*ΔR*/*R*, where *ΔR* is the change in resistance, *R* is the original resistance when unstretched) of PDSC E‐skin increased monotonously with the increase in tensile strain when the strain is <40%. Then the *ΔR*/*R* increased sharply until broken (Figure 4b). During the tension, the SIS fiber is stretched owing to the low modulus (≈1.7 MPa) while the stiff CB (modulus of ≈80 GPa) can hardly deform.^[^ ^59^ ^]^ The almost undeformed CB nanosheet will shift because of the large deformation of SIS fiber, leading to the increase of resistance (Figure 4c). Under small strains (1% to 5%), the *ΔR*/*R* of PDSC E‐skin showed stable and reproducible change with the strain changes (Figure 4d). Meanwhile, under large stains (10% to 40%), the *ΔR*/*R* of PDSC E‐skin remains the same pattern with the strain changes (Figure 4e). By fitting, a linear relationship between relative resistance changes and strain is obtained in the wide range of strain from 1% to 40%. The linear sensing response behavior is believed to be contributed from the elastic deformation of both the whole fibrous structure and the SIS fibers. When the strain is less than 40%, the fibrous structure bears most of the stretching energy and there is only a small deformation of SIS fiber. This can be proved by the gauge factor of 4.53 as the sensitivity mainly comes from the deformation of CB nanosheets, which resulted from the deformation of SIS fiber (Figure 4c,f). Therefore, as long as the fiber network structure is still able to deform and bear most of the energy input, the resistance change caused by SIS fiber deformation can be guaranteed to be linear. When the stain is over 40%, the SIS fiber will bear more deformation energy, thus leading to the high sensitivity (Figure 4b). However, the hard CB nanosheets will have an irreversible shift under large deformation, thus causing the baseline to drift (Figure S5, Supporting Information). More importantly, the PDSC E‐skin realizes the integration of wearable comfort functions through the soluble, crystalline hydrophobic fiber adhesive layer, including permeability, self‐adhesion, reversible adhesion, and water durability, which show superior properties to previous work (Table S1, Supporting Information).

Meanwhile, the excellent elasticity of the PDSC E‐skin makes the *ΔR*/*R* signal and the input strain match well. The waveform peaks of *ΔR*/*R* signal and strain synchronized, indicating negligible signal hysteresis under different stretching speeds (6–360 mm s^−1^) (Figure 4g). The *ΔR*/*R* signal remained consistent with the changed frequency tests under the strain of 20% and no electromechanical hysteresis was observed. The small mechanical hysteresis during the stretch and recovery guarantees the good repeatability of sensing (Figure 4h). Meanwhile, the response time of the PDSC to strain change is less than 160 ms owing to the excellent elasticity of fibrous structure and SIS rubbers (Figure 4i). The PDSC E‐skin exhibited excellent sensing stability in long‐time cycling tests. There is no obvious change of *ΔR*/*R* during 2000 cycles of 20% strain tests, indicating the remarkable durability and repeatability of the sensing function (Figure 4j).

**Figure 5:**
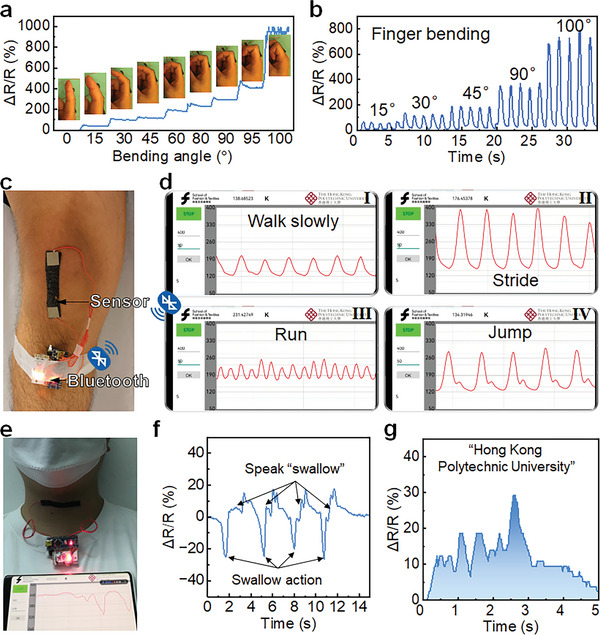
The relative resistance changes with a) continuous bending of finger and b) reciprocating bending of the finger. c) The PDSC E‐skin on the knee joint. d) The resistance changes on the smartphone during (I) slow walking, (II) stride, (III) running, and (IV) jumping. e) The photo of PDSC E‐skin on the throat. The relative resistance signals when f) swallowing and speaking the word “swallow” and g) speaking phrase “Hong Kong Polytechnic University”.

Based on the self‐adhesion and sensing functions of PDSC E‐skin, the wearable strain sensing was demonstrated with a microcircuit unit (MCU) and Bluetooth board (Figure S6, Supporting Information). The PDSC E‐skin was adhered to different parts of the body for real‐time monitoring of human activities. The mechanical adaptability allows PDSC E‐skin to discriminate large‐scale human motions like the movements of human joints. As depicted in **Figure** **5**a, the PDSC E‐skin was attached to a finger for bending monitoring. Under different bending angles of the finger and maintaining the bending state, the *ΔR*/*R* showed the relevant change and maintained the change as well (Figure 5a). The obvious *ΔR*/*R* change can be seen under 15°, even 5° bending angle changes. The *ΔR*/*R* was increased from 0 to ∼950% when the bending angle of the finger was increased from 0° to 100° gradually (Movie S1, Supporting Information). As the stretching of PDSC on the fingers comes from the elongation caused by bending rather than the direct stretching, the relationship between *ΔR*/*R* and bending angle is not linear. And the unusual peak shape when the finger is bent to 100° comes from the shaking due to the hard bending of the fingers to 100° (Figure 5a). Meanwhile, the quick response of PDSC E‐skin enables the detection of rapid and repeated bending of the fingers with the same *ΔR*/*R* signals as the maintaining the bending angle (Figure 5b; Movie S2, Supporting Information). Similarly, the PDSC E‐skin can remain firmly attached under drastic motions like running and jumping. Attaching the PDSC E‐skin to the knee joint (Figure 5c), the different motions can be monitored in real‐time on a smartphone by the MCU. The good sensitivity of the PDSC E‐skin strain sensor can distinguish the difference of *ΔR*/*R* signals under different motions (Figure 5d). With slow walking, the slow and small *ΔR*/*R* signal indicates the frequency of walking of ∼1 Hz and the small steps (Figure 5d‐I; Movie S3, Supporting Information). When walking with strides, the frequency remains the same of 1 Hz while the amplitude of *ΔR*/*R* signal was increased, indicating the large steps of walking (Figure 5d‐II; Movie S4, Supporting Information). While trotting in place, the frequency of *ΔR*/*R* signal was increased to ≈2 Hz, matching the frequency of running. And the amplitude of *ΔR*/*R* signal showed the low leg lift during running, demonstrating the fast but small steps (jogging) (Figure 5d‐III; Movie S5, Supporting Information). Meanwhile, during jumping, the *ΔR*/*R* signal showed clear leg bending during take‐off (large peak) and the bending of the leg for cushioning on landing (small peak) (Figure 5‐IV; Movie S6, Supporting Information). From the jumping signal, parameters such as idle time can be calculated for training guidance. From the *ΔR*/*R* signals, different actions can be clearly distinguished with parameters such as the waveform, frequency, and amplitude of the *ΔR*/*R* signals. Moreover, the high sensitivity of PDSC E‐skin allows tiny strain detection like the deformation of the throat during swallowing and talking (Figure 5e). When swallowing, the PDSC attached to the throat outputs a clear swallow action signal on time. The decrease of resistance of the swallow action signal shows the constriction and swallowing of the esophagus. Because of the self‐adhesion of PDSC, the constriction deformation can be clearly captured. When speaking, the throat will deform according to the syllables and speed of speaking. From the PDSC signals, three syllables of the word “swallow” have been shown and the repeated speaking of “swallow” shows the same pattern of resistance signals (Figure 5f). Besides, the PDSC E‐skin can distinguish the vibration accurately for long phrases. The *ΔR*/*R* signal during the talking of the phrase of “Hong Kong Polytechnic University” showed clear differences between different words (Figure 5g; Movie S7, Supporting Information). The above sensing results and the remarkable sensitivity and wide sensing range of the PDSC E‐skin strain sensors exhibit the potential of PDSC as a promising platform for wearable smart sensing.

### 2.4 The Wearability of PDSC E‐Skin

**Figure 6:**
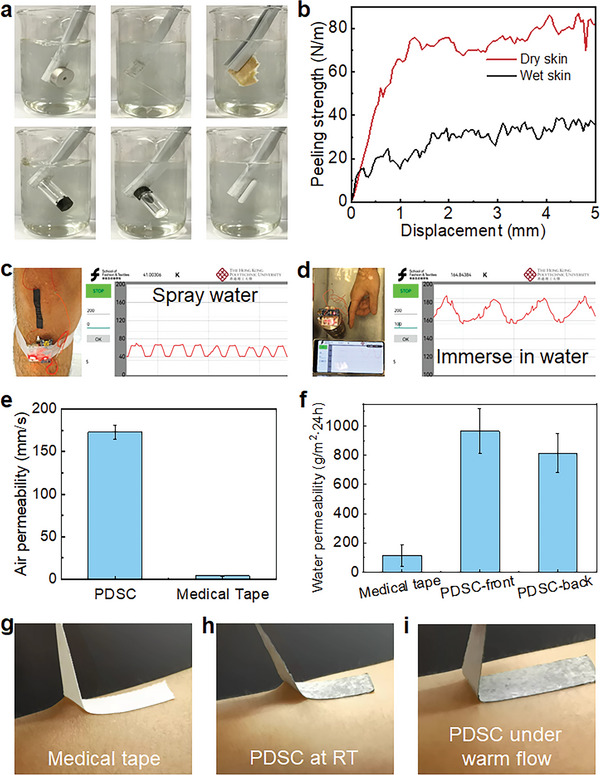
a) The underwater adhesion of PDSC E‐skin on metal, Teflon, glass, plastic, rubber, and pigskin. b) The peeling adhesion of PDSC on dry and wet skins. The resistance change signals of PDSC E‐skin c) with sprayed water and d) underwater. e) The air permeability and f) the water permeability of PDSC E‐skin compared to commercial medical tapes. The peeling process of g) medical tape, h) PDSC E‐skin at RT, and i) PDSC under warm flow on the skin.

As a skin‐attachable device, the wearability of PDSC E‐skin is critical for the applications, especially for long‐time wear and strenuous exercises.^[^ ^14^, ^33^ ^]^ Wearable comfort, such as the waterproofness, breathability and detachment‐on‐demand, gets more and more attention for the development of E‐skins. Since the E‐skin comes into direct contact with the skin, the PDSC E‐skin may encounter wet conditions like sweat secretion or external water from the environment. The hydrophobic PDMA adhesion layer endows the PDSC with good waterproof capabilities. The PDSC can maintain its robust adhesion underwater on different materials like metal, Teflon, glass, plastic, rubber, and pigskin (**Figure** **6**a). Therefore, the PDSC E‐skin can work under wet environments. The peeling strength of PDSC on wet skins remained ∼ 36 N/m, the half of adhesion on dry skins (Figure 6b). The wet adhesion allows PDSC attaching to wet skins, such as the monitoring of the state right after the exercise. We sprayed water on the PDSC E‐skin on the knee joint to simulate the sweat during the exercise (Figure 6c). The PDSC E‐skin can output the stable *ΔR*/*R* signal when walking (Figure 6c; Movie S8, Supporting Information). Moreover, the PDSC E‐skin can be used underwater as well. Immersing the finger with the PDSC attached in the water, the bending signal was detected as well and shown on the smartphone (Figure 6d; Movie S9, Supporting Information). The waterproof ability not only ensures motion sensing when sweating, but also provides the possibility to monitor water sports such as swimming. During the repeated tests underwater for 24 h, both the adhesion and sensing function remain stable (Figure S7, Supporting Information).

Another factor that affects wearable comfort is breathability, which includes both air permeability and water permeability. Good breathability can avoid the accumulation of sweat and adjust the temperature, thus supporting the long‐time and comfortable wearing.^[^ ^14^ ^]^ The fiber structures of both the adhesive layer and sensing layer endow the PDSC E‐skin with remarkable air breathability of 173.3 ± 8.3 mm s^−1^, which is ≈45 times higher than that of the commercial medical tape (Figure 6e). Meanwhile, the porous structures formed by fibers in both the adhesive layer and sensing layer can create capillary channels to fascinate the transfer of water from the skin. And the Janus PDSC with different hydrophobicity in the two layers can further improve the water permeability. The water molecules tend to transport from the hydrophobic side (adhesion layer) to the hydrophilic side (sensing layer) owing to the difference in the surface free energies.^[^ ^35^ ^]^ Therefore, the water permeability of PDSC E‐skin with the sensing layer on top reached 965.0 ± 153.9 g/m^2^▪24 h and with the adhesive layer on top reached 815.2 ± 132.9 g/m^2^▪24 h while the commercial medical tape only reached 115.1 ± 73.2 g/m^2^▪24 h (Figure 6f). The good air and moisture permeability guarantees wearable comfort for a long time.

The comfort during detachment of E‐skins after use is also one of the important considerations for wearability, especially on sensitive skins like baby skins, hairy skins, and injured skins.^[^ ^52^, ^60^ ^]^ There may be secondary damages like stripping, and skin tears caused by the strong adhesion E‐skins.^[^ ^33^ ^]^ The detaching‐on‐demand is critical after the use of E‐skin. Therefore, based on the crystallizable C18A segments in the PDSC adhesive layer, the adhesion can be switched off by thermal effect. Using a heating gun to heat the PDSC E‐skin can easily switch off the adhesion, thus achieving easy detachment (Movie S10, Supporting Information). Compared to the commercial medical tape and PDSC E‐skin at RT, the heated PDSC E‐skin could be peeled off from the skin without causing deformation of the skin (Figure 6g–i). Detaching‐on‐demand ensures the detaching comfort after use, which can extend the application in a wider range. The waterproof, breathability and controllable adhesion of PDSC E‐skin guarantee the wearable comfort in the whole process from use to the end.

Although there are E‐skins in previous literature that had better sensitivity, especially some electrospun nanofiber E‐skins,^[^ ^18^, ^22^, ^41^, ^42^, ^46^, ^61^ ^]^ there are still great challenges in functional integration, especially in the integration of wearable comfort functions.(Table S1, Supporting Information) The solid film E‐skins are usually limited in air/water permeability, which hinders long‐time wearing and sweaty conditions.^[^ ^20^, ^21^, ^22^, ^26^, ^28^, ^29^, ^30^, ^31^, ^51^, ^60^, ^62^ ^]^ The fibrous E‐skins bear the inherent permeability. However, the self‐adhesion is a challenge to integrate.^[^ ^18^, ^22^, ^35^, ^41^, ^42^, ^46^, ^61^ ^]^ On the basis of ensuring a certain sensing sensitivity, the PDSC E‐skin realizes the integration of wearable comfort functions through the soluble, crystalline hydrophobic fiber adhesive layer, including permeability, self‐adhesion, reversible adhesion, and water durability.

## 3 Conclusion

In conclusion, the new Janus PDSC E‐skin prepared by electrospinning with PDMA adhesive layer and SIS/CB sensing realized self‐adhesive, detach‐on‐demand, breathable, and waterproof capabilities for strain sensing. The PDMA adhesive layer with crystallizable C18A segments endows the PDSC E‐skin with not only strong adhesion, but also the detaching‐on‐demand ability by thermal. The strong shear and peeling adhesion of PDSC E‐skin of ∼520 kPa and ∼700 N/m is much higher than the commercial 3M tapes, ensuring stability and durability during wearing. Meanwhile, the switch‐off shear and peeling adhesion by thermal ≈50 °C was decreased to ≈120 kPa and ≈85 N m^−1^, respectively, realizing the easy and painless detaching after use. During the wear, the hydrophobicity of the PDMA adhesive layer can prevent the water from disabling the adhesion, extending the application scenarios like wet environments. Meanwhile, the micro‐fiber structure of the whole PDSC endows the E‐skin with air breathability and water permeability, which are further enhanced by the Janus hydrophobicity of PDMA layer and SIS/CB layer. With a Bluetooth MCU, both the drastic motions like exercise and tiny deformations like the throat deformation by speaking can be monitored real‐timely. The PDSC E‐skin with self‐adhesive, detach‐on‐demand, breathable, and waterproof abilities can be applied in areas like long‐time exercises for motion monitoring and human‐machine interfaces.

Meantime, there are some limitations of current work in sensitivity and breathable packaging. The sensing functions of PDSC have a gap compared to some previous works. The specialized crack design for the sensitivity of these works gives good inspiration for future studies. Meanwhile, the sensing function can be interfered by water. The sensitivity of the signal is reduced, and some signal details are lost as the conductivity of water. The breathable package of E‐skins to prevent the water from interfering the electrical signals will be the further research direction.

## 4 Experimental Section

### Materials and Equipment

The styrene‐isoprene block copolymer (SIS) rubber was purchased from SINOPEC Baling Petrochemical Co., Ltd., China. Carbon black (CB) nanosheets, N, N‐dimethylacrylamide (DMA), n‐octadecyl acrylate (C18A), lauryl methacrylate (C12M), 2‐Hydroxy‐4′‐(2‐hydroxyethoxy)−2‐methylpropiophenone (2959), Dimethyl Formamide (DMF) and ethyl acetate (EA) were purchased from DIECKMANN, China. The double‐layer tape and medical tape were purchased from 3M corporation, US.

The SEM image and EDS tests of PBIA were conducted by SEM (Tescan MIRA). The tensile tests, shear adhesion, peeling adhesion, and tensile strain sensing were conducted by the universal testing machine (5566, Instron). The DSC tests were conducted by DSC 8000, PerkinElmer. Small‐angle X‐ray scattering measurement was tested on the X‐ray Diffractometer (Rigaku SmartLab 9 kW). The water contact angles were tested on the SDC‐350, Dynetech. The resistance change during tension was tested by a 2010 multimeter, Keithley. The warm flow was generated from a heating gun for adhesion switching. The air permeability was tested by the Air Permeability Tester (SDL International M021S).

### Fabrication of PDSC E‐Skin

The fabrication process of PDSC is illustrated in Figure 1. The SIS was dissolved in EA with a concentration of 0.25 g ml^−1^. The CB was dispersed in the EA/DMF (1:1 volume) with the concentration of 0.02 g ml^−1^. Then both solutions were poured into an injection syringe (needle: 14G) and electrospun on the silicone paper at the same time. The voltage, feed rate, needle distance, and scan rate of SIS solution were set to −2/13 kV, 4 ml h^−1^, 100 mm, and 20 mm s^−1^, respectively. The voltage, feed rate, needle distance, and scan rate of CB solution were set to −5/10 kV, 1 ml/h, 150 mm, and 20 mm s^−1^, respectively. After electrospinning, the film was dried in the oven at 60 °C for 12 h and then attached with conductive cloth tape as the electrode. Meanwhile, the PDMA solution was prepared by mixing DMA, C18A, C12M, 2959 with the mole ratio of 60:25:15:1 and adding EA to prepare the solution with 50% concentration in volume. Then the solution was polymerized under 365 nm UV for 12 h. After polymerization, the PDMA solution was electrospun on the SIS/CB layer with the voltage, feed rate, needle distance, and scan rate of CB solution as −2/13 kV, 3 ml h^−1^, 150 mm and 20 mm s^−1^, respectively. After electrospinning of PDMA layer and drying in the oven at 50 °C, the PDSC E‐skin was prepared.

### Adhesion Test of PDSC E‐Skin

The shear adhesion was tested by two metal plates with 20 mm × 10 mm PDMA adhesive layer sandwiched. The 50 °C temperature was realized by a semiconductor heater attached to one metal plate, which can maintain the constant temperature at 50 °C. The adhesive was heated to 50 °C and pressed on the metal plate. The universal testing machine with a 50 kN load cell was used to test the shear adhesion on (with the adhesive cooling down to RT) and shear adhesion off (keeping the adhesive 50 °C) with a shearing speed of 1 mm s^−1^. The double‐side tape was tested with the same procedure.

The peeling adhesion was tested on the PET film with the universal testing machine. An electric heater was used to heat the PET substrate for peeling adhesion on/off tests. The PDSC E‐skin with aluminum foil attached to the sensing layer and 3M commercial medical tape was cut into 10 mm × 120 mm size strips for the peeling tests. The PET substrate was heated to 50 °C and the PDSC E‐skin and medical tape were on. The universal testing machine with a 50 kN load cell was used to test the peeling adhesion on (with the adhesive cooling down to RT) and peeling adhesion off (keeping the adhesive 50 °C) with a peeling speed of 1 mm s^−1^. The on‐body adhesion was tested by pressing the heated PDSC E‐skin (≈50 °C) to the skin until cooling to RT. Then the peeling, sensing, underwater tests, and other adhesion tests were conducted.

### The Tests of Sensing

The performance of resistance strain sensor was tested by the universal testing machine with a multimeter. Different strains from 1% to 50% were applied and the relative resistance was recorded. Meanwhile, different frequencies from 0.008 to 0.5 Hz were applied to test the response speed of PDSC E‐skin. The 2000 cycles of tensile tests with 20% strain were conducted to test the durability of PDSC E‐skin. For body motion sensing, a home‐built Bluetooth MCU was used to test the resistance change during the motion and transmit the resistance data to the smartphone by Bluetooth. A smartphone app was used to record and show the resistance real‐timely during the motion. The PDSC E‐skin was heated by the warm flow to ≈50 °C and pressed on the skin until cooling to RT. Then the sensing under different motions was tested. Testing the wearables was carried out with the assistance of a human participant volunteer (an author of this article), and informed written consent was obtained from the participant.

### Permeability Tests

The air permeability was tested by Air Permeability Tester (SDL International M021S) with a pressure of 125 Pa. The water permeability was tested according to the standard (GB/T 12704.2‐2009). A 100 ml beaker with ≈50 ml water was used for water permeability tests. The samples were covered on the rims with tape. Before the tests began, the total weight of the whole device was measured as *m*_0_. The device was placed in the chamber with ≈20 °C and ≈65% RH environment for 12 h. Then the weight of the whole device was measured as *m*_1_. The final water permeability (*WP*) was then calculated by the following equation:

(3) $$WP=\frac{m_{0}-m_{1}}{A\times t}\;$$

where *A* is the sample area and *t* is the testing time.

## Conflict of Interest

The authors declare no conflict of interest.

## Supporting information

Supporting Information

Supporting Information

Supporting Information

Supporting Information

Supporting Information

Supporting Information

Supporting Information

Supporting Information

Supporting Information

Supporting Information

Supporting Information

## Data Availability

The data that support the findings of this study are available from the corresponding author upon reasonable request.
