# Supplementary material for: Smart‐Adhesive, Breathable and Waterproof Fibrous Electronic Skins
Source: Adv Sci (Weinh). 2024 Jul 25;11(36):2405828. doi: 10.1002/advs.202405828 (PMC11423153; doi:10.1002/advs.202405828)
Supplement: Supplementary file 1 — Supporting Information [file ADVS-11-2405828-s010.docx]

**Supporting Information**

**Caption of supplementary movies**

Supplementary movie S1: The real-time sensing of PDSC E-skin for constant bending of the finger.

Supplementary movie S2: The sensing of PDSC E-skin for cyclic finger bending. The video is speed up by 3 times.

Supplementary movie S3: The sensing of PDSC E-skin on the knee joint for slow walking.

Supplementary movie S4: The sensing of PDSC E-skin on the knee joint for stride.

Supplementary movie S5: The sensing of PDSC E-skin on the knee joint for running.

Supplementary movie S6: The sensing of PDSC E-skin on the knee joint for jumping.

Supplementary movie S7: The sensing of PDSC E-skin on the throat for swallowing and speaking.

Supplementary movie S8: The sensing of PDSC E-skin on the knee joint for walking with sprayed water.

Supplementary movie S9: The sensing of PDSC E-skin for finger bending underwater.

Supplementary movie S10: The peeling of medical tape, PDSC at adhesion on and off state.


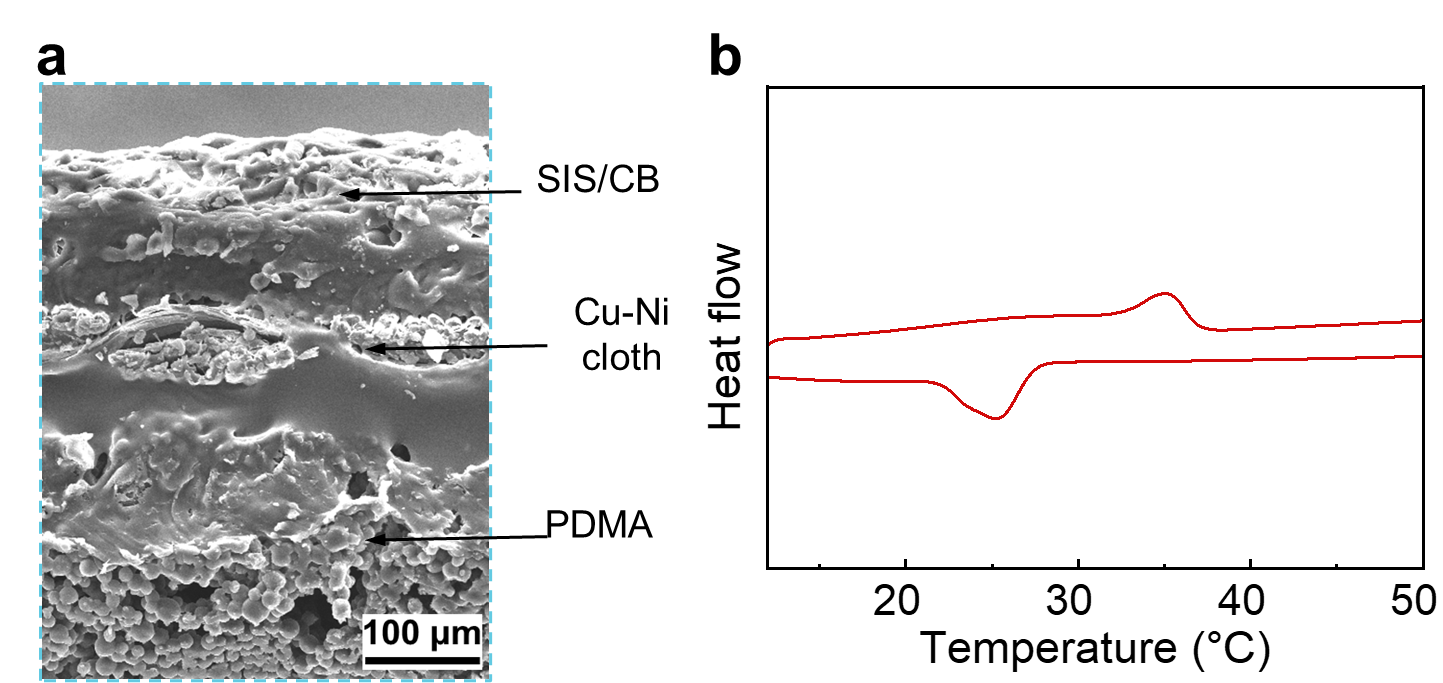


**Figure S1.** (a) The cross-section of PDSC at the position of the electrode. (b) The DSC scan of PDMA solid film.


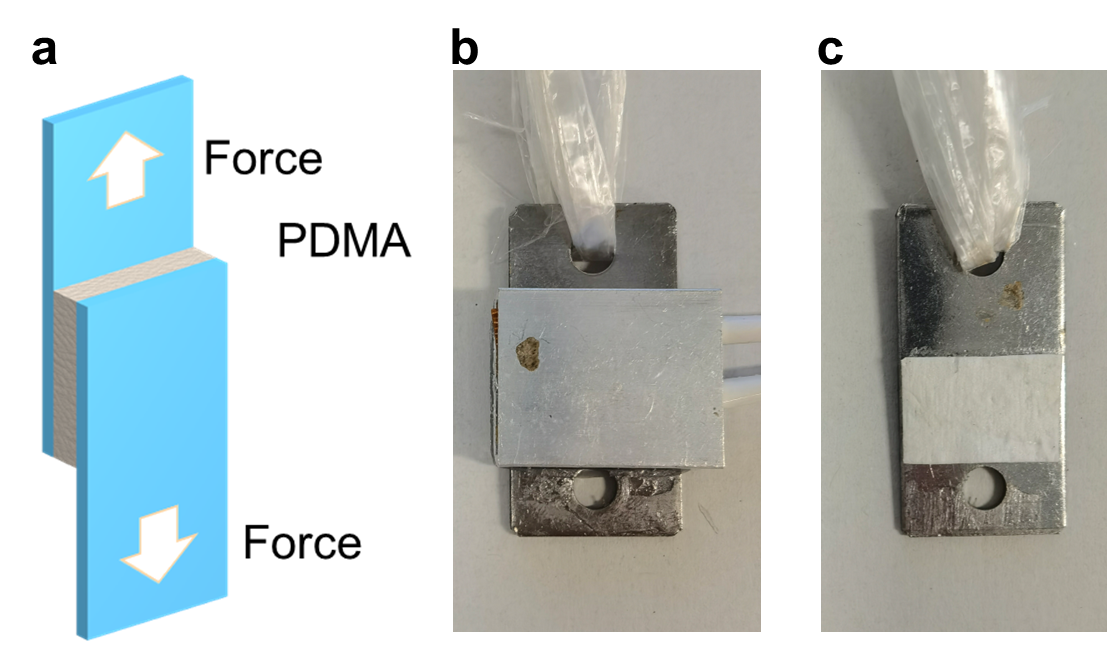


**Figure S2.** (a) Illustration of the shear adhesion tests. (b)semiconductor heater for shear adhesion tests. (c) The PDMA layer on the metal plate.


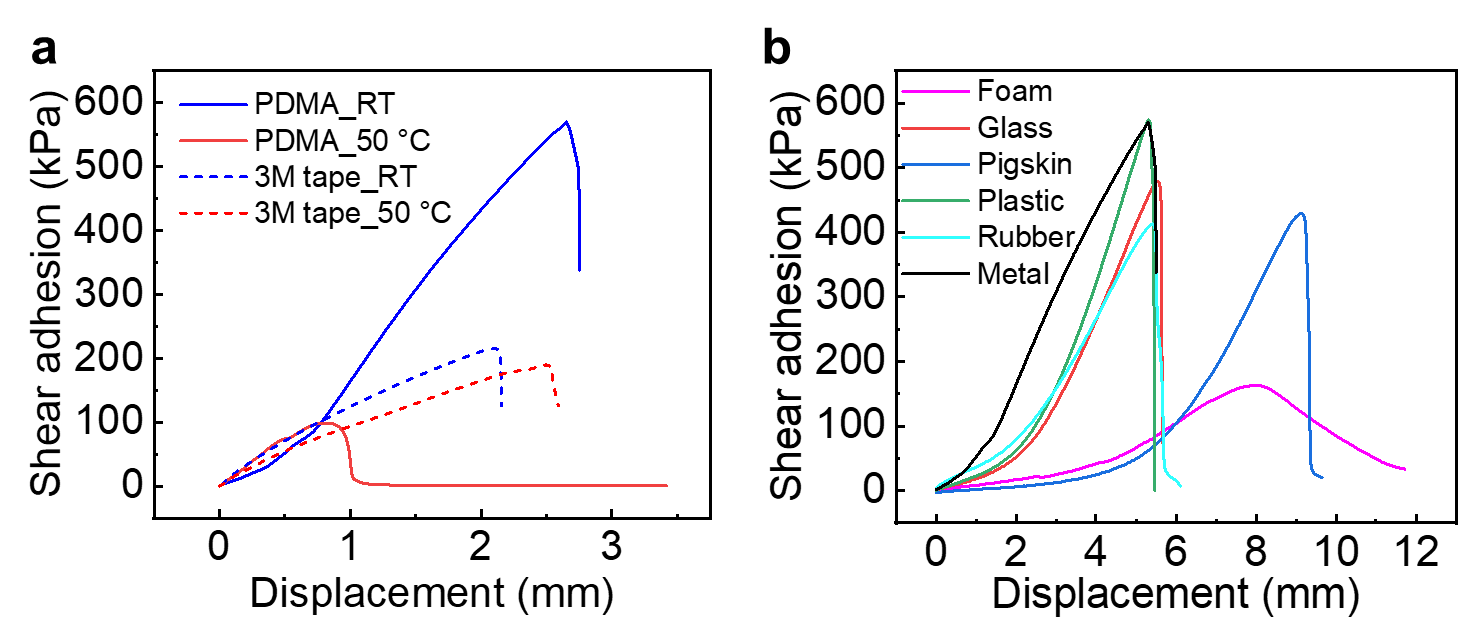


**Figure S3.** (a) The typical shear adhesion curve of PDSC and 3M double-side tape on steel at different temperatures. (b) The typical shear adhesion curve of PDSC on different substrates.

**
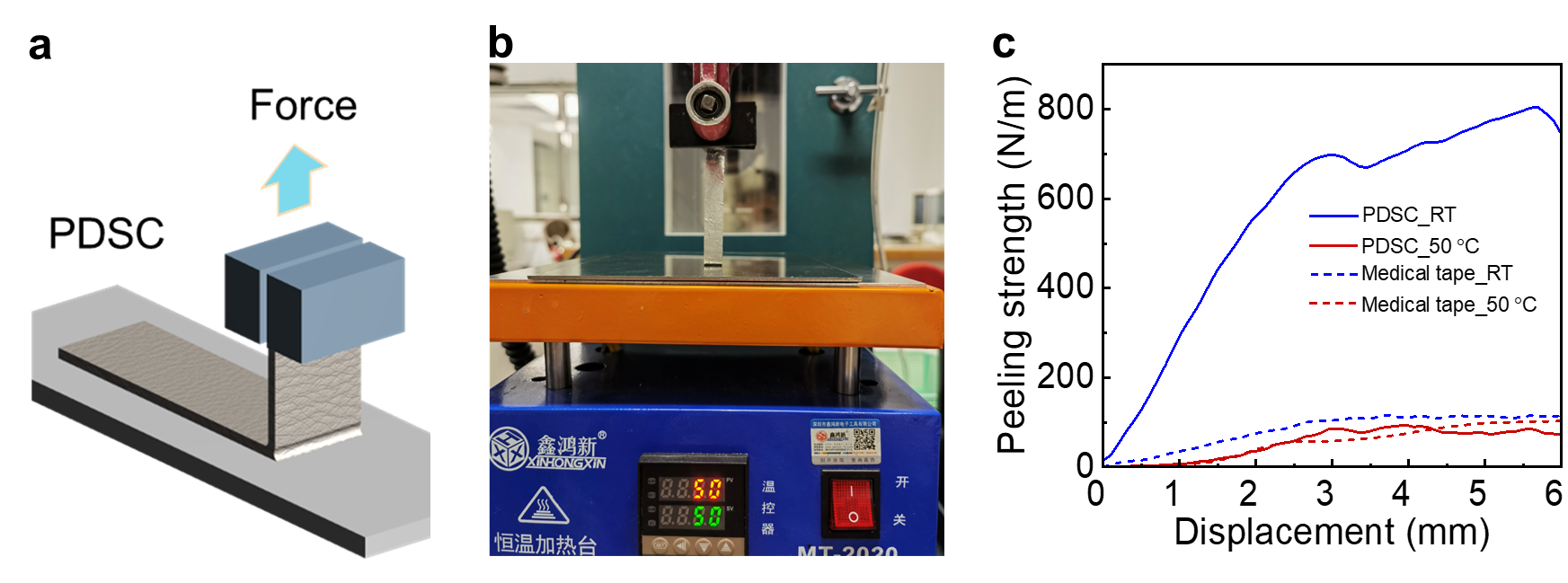
**

**Figure S4.** (a) Illustration of the peeling test of PDSC E-skin. (b) The electric heater for peeling adhesion tests. (c) The peeling curve of PDSC and 3M medical tape.


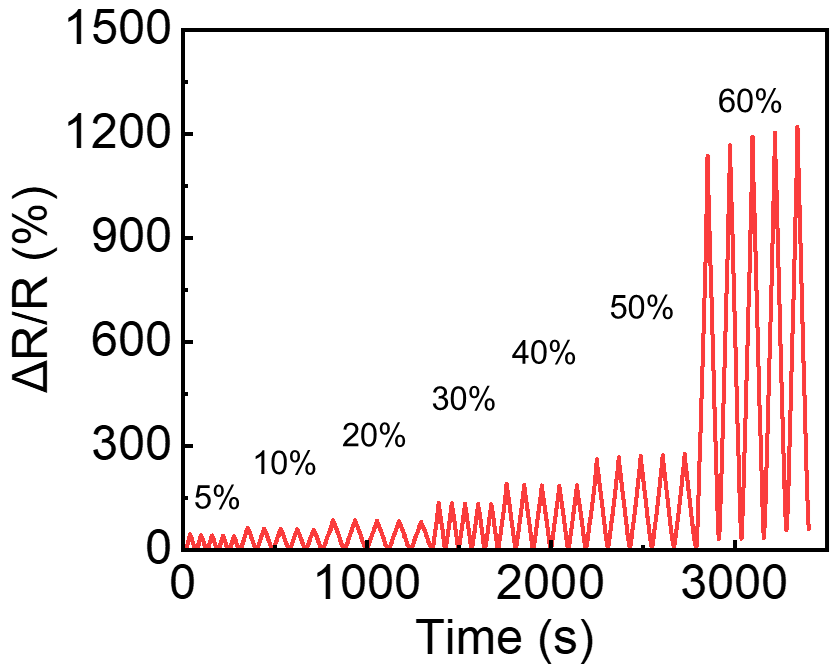


**Figure S5.** The large strain sensing test and relevant ΔR/R signals.


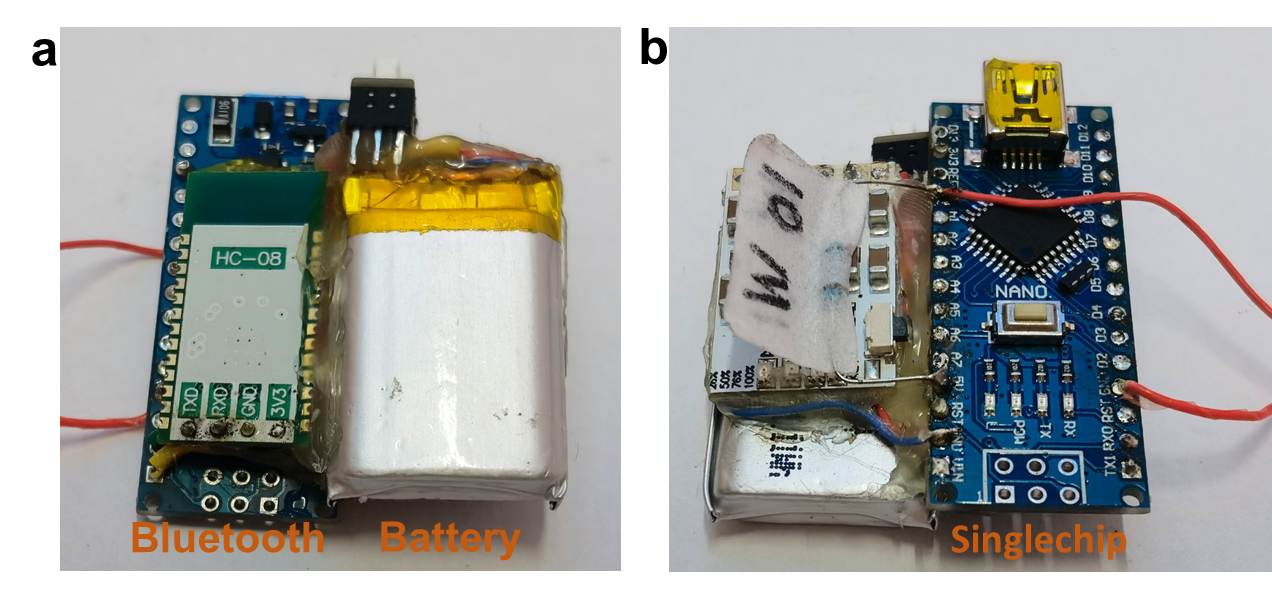


**Figure S6.** (a) The front side and (b) back side of the tailor-made MUC with Bluetooth.


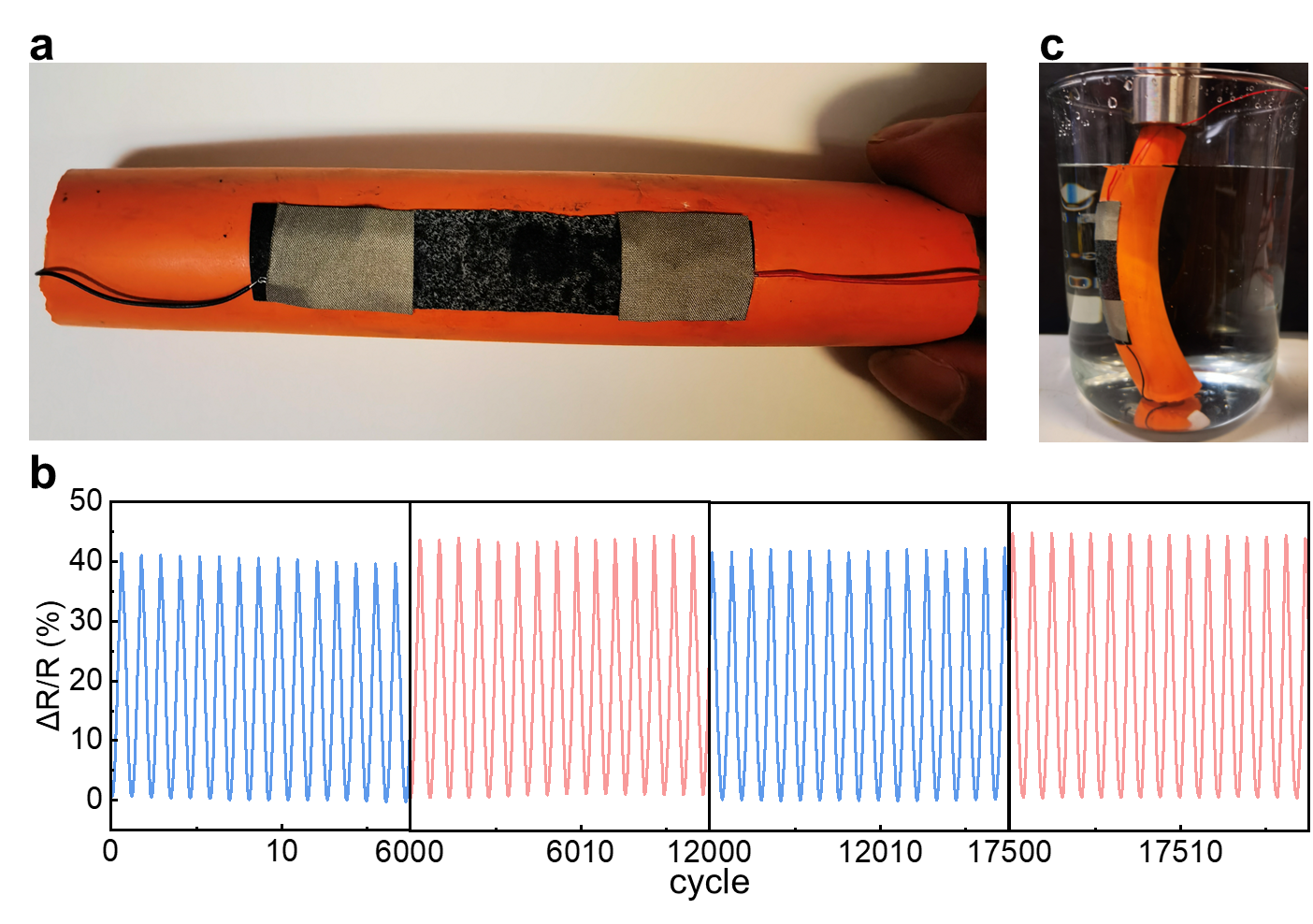


**Figure S7.** (a) The PDSC is attached to a rubber tube for bending tests. (b) The durability of PDSC E-skin underwater. (c) The photo of PDSC after the 24-hour test.

**Table S1.** Comparison of wearable functions of PDSC and previous works. (IL means ionic liquid, the unit of shear adhesion and normal adhesion (with *) is kPa, the unit of peeling adhesion is N/m)

| Material | Function | Breatha-bility | Self-adhesion | Smart adhesion | Water  durability | Ref |
| --- | --- | --- | --- | --- | --- | --- |
| PU-CNT/RGO film | Strain sensing | − | 130 kPa | − | − | ^[1]^ |
| Alglnate hydrogel | Strain sensing | − | 537.4 N/m | − | 649.3 N/m | ^[2]^ |
| WPU hydrogel | Strain sensing | − | 59.6 kPa | − | − | ^[3]^ |
| Hydrogel | EEG | − | 59.7 N/m | − | 44.1 N/m | ^[4]^ |
| HAPN/PU-Ag nanofiber | EMG | 1.7 kg/m^2^/day | 43.1 N/m | − | − | ^[5]^ |
| CNF/PBA-IL hydrogel | Strain sensing | − | 273.5 N/m | − | − | ^[6]^ |
| Elastomer-IL | Strain sensing | − | 33.1 N/m | √ | √ | ^[7]^ |
| Glycerol hydrogel | Strain sensing | − | 12.6 kPa | − | − | ^[8]^ |
| PU-MXene nanofiber | Strain sensing | 2.2 kg/m^2^/day | √ | − | − | ^[9]^ |
| TPU-MXene/CNT nanofiber | Strain sensing | √ | 145 N/m | − | 145 N/m | ^[10]^ |
| Elastomer | Strain sensing | − | 300 kPa* | √ | 250 kPa* | ^[11]^ |
| Elastomer/IL | Strain sensing | − | 27.7 kPa | − | √ | ^[12]^ |
| PAA hydrogel | Strain sensing | − | 30.2 kPa | − | − | ^[13]^ |
| Chitin hydrogel | Strain sensing | − | 113.2 kPa | − | − | ^[14]^ |
| TPU-CNT nanofiber | Strain sensing | 3 kg/m^2^/day | − | − | − | ^[15]^ |
| Elastomer/IL | Strain sensing | − | 90N/m | √ | 70N/m | ^[16]^ |
| SIS-CB/ Polyacrylate nanofiber | Strain sensing | 0.97 kg/m^2^/day | 78N/m | √ | 35N/m | This work |

**References**

[1] S. Wang, Y. Fang, H. He, L. Zhang, C. A. Li, J. Ouyang, *Adv. Funct. Mater.* **2021**, *31*, 2007495.

[2] B. Ying, R. Z. Chen, R. Zuo, J. Li, X. Liu, *Adv. Funct. Mater.* **2021**, *31*, 2104665.

[3] H. Wang, X. Li, Y. Ji, J. Xu, Z. Ye, S. Wang, X. Du, *J. Mater. Chem. B* **2022**, *10*, 2933.

[4] G. Yang, K. Zhu, W. Guo, D. Wu, X. Quan, X. Huang, S. Liu, Y. Li, H. Fang, Y. Qiu, Q. Zheng, M. Zhu, J. Huang, Z. Zeng, Z. Yin, H. Wu, *Adv. Funct. Mater.* **2022**, *32*, 2200457.

[5] X. Yang, S. Wang, M. Liu, L. Li, Y. Zhao, Y. Wang, Y. Bai, Q. Lu, Z. Xiong, S. Feng, T. Zhang, *Small* **2022**, *18*, 2106477.

[6] X. Yao, S. Zhang, L. Qian, N. Wei, V. Nica, S. Coseri, F. Han, *Adv. Funct. Mater.* **2022**, *32*, 2204565.

[7] Q. Zeng, F. Wang, R. Hu, X. Ding, Y. Lu, G. Shi, H. Haick, M. Zhang, *Adv. Sci.* **2022**, *9*, 2202635.

[8] H. Zhou, J. Lai, B. Zheng, X. Jin, G. Zhao, H. Liu, W. Chen, A. Ma, X. Li, Y. Wu, *Adv. Funct. Mater.* **2022**, *32*, 2108423.

[9] T. Cui, Y. Qiao, D. Li, X. Huang, L. Yang, A. Yan, Z. Chen, J. Xu, X. Tan, J. Jian, Z. Li, S. Ji, H. Liu, Y. Yang, X. Zhang, T.-L. Ren, *Chem. Eng. J.* **2023**, *455*, 140690.

[10] Y. Hao, Q. Yan, H. Liu, X. He, P. Zhang, X. Qin, R. Wang, J. Sun, L. Wang, Y. Cheng, *Adv. Funct. Mater.* **2023**, *33*, 2303881.

[11] D. Tan, F. Meng, Y. Ni, W. Sun, Q. Liu, X. Wang, Z. Shi, Q. Zhao, Y. Lei, S. Luan, L. Xue, *Chem. Eng. J.* **2023**, *471*, 144625.

[12] H. Wang, Y. Mao, D. Ji, L. Wang, L. Wang, J. Chen, X. Chang, Y. Zhu, *Chem. Eng. J.* **2023**, *471*, 144674.

[13] Y. Wang, Y. Yu, F. Zhao, Y. Feng, W. Feng, *Adv. Compos. Hybrid Mater.* **2023**, *6*, 65.

[14] J. Zhang, Y. Hu, L. Zhang, J. Zhou, A. Lu, *Nano-Micro Lett.* **2023**, *15*, 8.

[15] Y. Tian, M. Huang, Y. Wang, Y. Zheng, R. Yin, H. Liu, C. Liu, C. Shen, *Chem. Eng. J.* **2024**, *480*, 147899.

[16] D. Tan, B. Xu, K. Y. Chung, Y. Yang, Q. Wang, Y. Gao, J. Huang, *Adv. Funct. Mater.* **2023**, 2311457.
